# An investigation of urban pedestrian behaviour in Bangladesh using the Perceptual Cycle Model

**DOI:** 10.1016/j.ssci.2021.105214

**Published:** 2021-06

**Authors:** Mithun Debnath, Shahnewaz Hasanat-E-Rabbi, Omar Faruqe Hamim, Md. Shamsul Hoque, Rich C. McIlroy, Katherine L. Plant, Neville A. Stanton

**Affiliations:** a Department of Civil Engineering, Ahsanullah University of Science & Technology, 141 & 142, Love Road, Tejgaon Industrial Area, Dhaka-1208, Bangladesh; b Accident Research Institute, Bangladesh University of Engineering and Technology, Dhaka-1000, Bangladesh; c Department of Civil Engineering, Bangladesh University of Engineering and Technology, Dhaka-1000, Bangladesh; d Human Factors Engineering, Transportation Research Group, University of Southampton, Southampton, UK

**Keywords:** Perceptual Cycle Model, Think-aloud, Decision-making, Pedestrian behaviour, Collision, Low-middle income country

## Abstract

In Bangladesh, pedestrians remain the most neglected road user group in terms of research and safety measures, even though they are involved in nearly 50% of all fatal collisions. In the Dhaka metropolitan area, this statistic rises to around 65%. To reduce the recurrence of such collisions, it is necessary to understand the underlying thought processes of pedestrians, and how pedestrians interact with different road users, particularly when crossing the road. This study contributes to knowledge through the analysis of verbal data from 46 pedestrians, framed in terms of the Perceptual Cycle Model, where cognitive processes are described in terms of three categories: schema (mental templates), action (doing things), and world (environmental information). Concurrent verbal reports were provided by participants while they negotiated a busy area of mixed traffic in the centre of Dhaka city. The analysis revealed some of the factors that affected decision-making (for road crossing behaviour) at the different road sections. Many external factors (e.g., street sellers, rubbish blocking the path, lack of shade and poor pavement condition) prevented the pavement from being used and contributed to riskier road crossing behaviour. Some safety implications and related recommendations are presented.

## Highlights

• Analysing pedestrian decision-making using Perceptual Cycle Model in urban context.
• Past experience, action and environment shapes decision-making of pedestrians.
• Discovered the difficulties experienced by pedestrians during navigation.
• Proposing recommendations to improve pedestrian safety in a low-income setting.

## 1 Introduction

The problem of pedestrian death and injury due to road traffic collisions is acute (WHO, 2018). Across a wide variety of nations, vulnerable road users, in particular pedestrians, are found to be over-represented in road traffic casualty and fatality statistics (e.g., Afukaar et al., 2003, Hijar et al., 2003, Ariffin et al., 2010, Mabunda et al., 2008, Mohan et al., 2009, Naci et al., 2009, Zegeer and Bushell, 2012). This problem is especially acute in low- and middle-income countries (WHO, 2018). In Bangladesh, a least developed country (OECD, 2019), collisions involving pedestrian account for between 37% and 73% of all collisions in metropolitan areas, and around 56% in non-metropolitan areas. It has also been found that 41% of collisions involving pedestrian occur during the crossing of roads and 39% occur while walking on the road (Mahmud et al., 2009).

Various studies have been carried out to help understand the factors contributing to pedestrian collisions, with pedestrian behaviour being a major focus. For instance, Lenard and Hill (2004) studied the causal factors of such collisions in the UK using an on-the-spot collision investigation database. From their study, it was found that, among various factors, road environmental conditions (e.g., obstruction from parked car, vegetation, road slope, road surface, etc.) and ‘failed to look’ behaviours accounted for around 30% of collisions, followed by ‘inattention’ (24%), ‘looked but did not see’ and ‘in a hurry’ (19%), and ‘failed to judge other’s path or speed’ (18%). Tulu et al. (2013) investigated the effect of various factors in pedestrian collision causation in Ethiopia, identifying various risky pedestrian behaviours such as walking at night, walking on roads, and illegal crossing as significant contributors to pedestrian fatalities.

In low-income countries (like Bangladesh), two significant challenges for road safety include a lack of research funding and the underreporting of collisions (Hoque et al., 2007a). For example, the Accident Research Institute, a Dhaka-based organisation, showed a large mismatch between the road traffic collisions reported by the police and those reported in the Microcomputer Accident Analysis Package version 5 (MAAP5) database, a software developed by Transport Research Laboratory (TRL), UK, and used by the Accident Data Units (ADU) of police to keep records of road traffic collision database. This mismatch is due to inconsistencies and inaccuracies in completing the forms used by police at the scene of a collision, inaccurate data transcription from those forms to the MAAP5 database, and a lack of trained officers for data entry. Indeed, police do not always investigate collisions for which some roadside reporting has been performed, hence there are fewer collisions recorded in the MAAP5 database than there are in the record of police report forms. For example, Mahmud (2019) found that nearly one-third of reported collisions do not appear in the MAAP5 database. It is worth noting that, in Bangladesh, the police force represents the authorized government organisation for collecting and disseminating road traffic collision data, and there is no hospital nor insurance-based data collection system. Daily newspapers also represent a source of collision data; however, the main problem of using newspaper data is that they are not consistent, lack good quality data, and show an under-reporting of rural or non-fatal collisions (Hoque et al., 2007b). As a result, these data are not always useful for research purposes.

In Bangladesh, few research efforts have been undertaken to explore the factors contributing to road traffic collisions. There exists limited work on the development of a collision map or database using a GIS tool (Ahmed, 2012), while other researchers have provided an initial methodological analysis (Nury et al., 2012). Recently, some research has emerged that takes a sociotechnical approach to the investigation of road traffic collisions (Hamim et al., 2020); however, very little research work on pedestrian safety can be found in the context of Bangladesh. This is the focus of the current work.

To ensure the safety of pedestrians, it is necessary to understand their behaviour and decision-making processes. In this context, McIlroy et al., 2019, McIlroy et al., 2020) explored the effects of attitudes towards road safety and demographic characteristics on respondents’ self-reported pedestrian behaviours, finding links between the studied factors; however, to the authors’ knowledge, no other work related to pedestrian psychology in Bangladesh has previously been performed.

Different road users have different needs when interacting with the road system, hence their perceptions regarding the system are different. Perceptions develop from road-use experience and road design (Salmon et al., 2013a), hence, different road users perceive the same situation differently. For example, a driver may expect that pedestrians will stop when they give a signal to stop, whereas a pedestrian’s previous experience may encourage them to cross the road by thinking that the driver will stop for them. Such a situation could give rise to conflict between the driver and the pedestrian. This situation could also be true for other road user conflicts, for example drivers versus motorcyclists and cyclists. Salmon et al. (2014) found that drivers do not always look out for motorcyclists and cyclists as their presence was not usually expected. Those authors described the identified differences in experiences and expectations between road user groups using schema theory and the Perceptual Cycle Model (PCM), with the underlying theory pointing to a mismatch between different road users’ situation awareness in similar road contexts. This mismatch, or incompatibility, may also be present between pedestrians and other road users, hence the ‘looked-but-failed-to-see’ errors leading to collisions.

This paper explores the perceptions and decision-making processes of pedestrians, using the information processing framework of the PCM (see Section 2) (Neisser, 1976). Previous research has applied this model in the road safety domain by studying the decision-making processes of car drivers and cyclists (Salmon et al., 2014) and extensively to the study of decision-making in aviation (Plant and Stanton, 2016). However, to our knowledge this methodology has not yet been used to explore pedestrian decision-making, nor has it been applied in the context of a low-income country. In the road safety domain, most of the research carried out in low-income setting follows traditional approaches that focus on short-term collision mitigation solutions. For example, one popular tool is the Road Safety Audit (RSA). This focusses on hazardous location identification, with attention directed to the physical factors (e.g., road geometries) that could contribute to collisions. Although of some use, RSA does not deal with enforcement issues or road user behaviours. For example, if there is an overpass or underpass at a given location, RSA may aid in the identification of structural safety issues related to, e.g., slippery surfaces, slopes, or step height differences. However, the rationale of use or non-use of that overpass or underpass, a significant issue in many LMICs (e.g., Hasan et al. 2020) is out of scope of RSA. Similarly, for footpaths, RSA may identify some hazards (regarding, e.g., the height of the footpath from the road, manhole covering locations, kerbs, and gutter designs etc.), but factors such as the presence of temporary shops on the footpath, or the lack of enforcement on parking (and its impact on pedestrian movement), are beyond the scope of RSA. As such, behavioural studies aimed at improving our understanding of how and why pedestrians make certain decisions on the road are necessary. Applying the PCM to the verbal reports of pedestrians provided while interacting with the road system in a naturalistic way, represents one such as-yet under-explored avenue.

## 2 The Perceptual Cycle Model

**Fig. 1:**
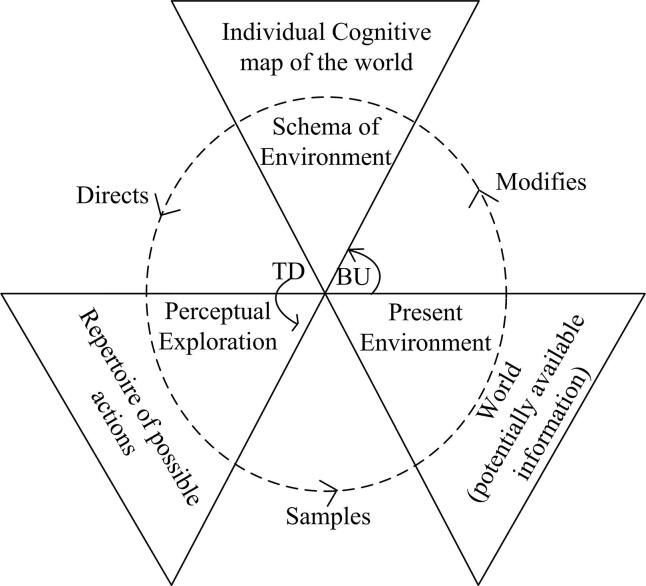
Perceptual Cycle Model (Neisser 1976).

It has been argued that Neisser’s (1976) perceptual cycle model (Fig. 1) underpins road user situation awareness (Plant and Stanton, 2013). The model’s main thesis is that the processing of information occurs in a reciprocal, cyclical manner (Walker et al., 2011, Plant and Stanton, 2016). The PCM posits that individuals hold mental templates about the world; this comes from past experience and is termed ‘schema’ in the language of the PCM. It explains that when a person experiences a similar situation to one experienced previously, they interact with the current environment in a manner similar to how they interacted with the previously experienced, similar environment. During interaction, their mental template, or schema, of the situation gets modified based on the environmental information present, thus changing their interaction with the situation (Salmon et al., 2014), and modifying their perception of that situation (Plant and Stanton, 2016). This modification is a cyclical process rather than linear, and active rather than passive (Walker et al., 2011). According to the PCM, information flow is both top down (TD) and bottom up (BU) (Plant and Stanton, 2016). In the top-down (TD) process, an individual’s action is affected by the schema one holds, i.e., schema directs or modifies the action. In bottom-up (BU), world environment and information modify the schema of an individual. This modified schema then affects the action of the pedestrian. Schema driven, TD information processing is shown on the left side of the PCM diagram, and environment driven, BU processing is shown on the right side of the diagram.

Understanding the way information flows through the PCM can shed light on the decision-making processes of the pedestrians, and it is an approach that has seen some attention in the road safety domain. For example, Salmon et al. (2013b) used the PCM in their analysis of a collision between a semi-trailer truck and a train at a level crossing. From their study, the primary contributing factor contributing to the collision was found to be the faulty activation of schema due to a ‘looked-but-failed-to-see’ error, an error that led to erroneous decision-making. Banks et al. (2018) also used the PCM to explore the circumstances surrounding a fatal collision involving a Tesla with its autopilot feature engaged. The car’s driver was argued to have been at fault in the National Highway Traffic Safety Administration’s preliminary investigation; however, the PCM findings emphasized the role of the vehicle’s designers, pointing to faults in the autopilot features that gave rise to the driver’s erroneous decision-making. In all examples, the PCM analysis supported further insight into the collisions analysed.

Against this background, the aim of the current research is to use the PCM to help understand urban pedestrian behaviour in Bangladesh, a low-income, least developed country, and what this might mean for traffic safety intervention design. Such understanding of behavioural and decision-making phenomena in a complex road traffic environment can aid in designing urban road features for improving pedestrian safety especially in low-income settings.

## 3 Method

### 3.1 Participants

To assess the utility of the PCM in revealing insights into pedestrian decision making and situation awareness in Dhaka, Bangladesh, a total of 46 participants who were familiar with or living in the vicinity of the study location (described below), or who interacted with the intersection at that location on a regular basis were recruited. Due to resource constraints, a convenience sample was sought, hence many participants were university students. Although not representative of the wider Dhaka population, many educational institutions (four schools and colleges, one university, and more than twenty different coaching centres) are present within the study area, hence the sample largely reflects the on-road reality (we discuss this further in the discussion section). The education levels of the participants varied from secondary school certificate (SSC) to Master of Science (MSc), with some of the participants being employed, others being students. Of the 46 participants who completed the study, 36 were male and 10 were female. The age of the participants ranged from 19 to 58 (mean = 26.96, SD = 10.34).

### 3.2 Study route

**Fig. 2:**
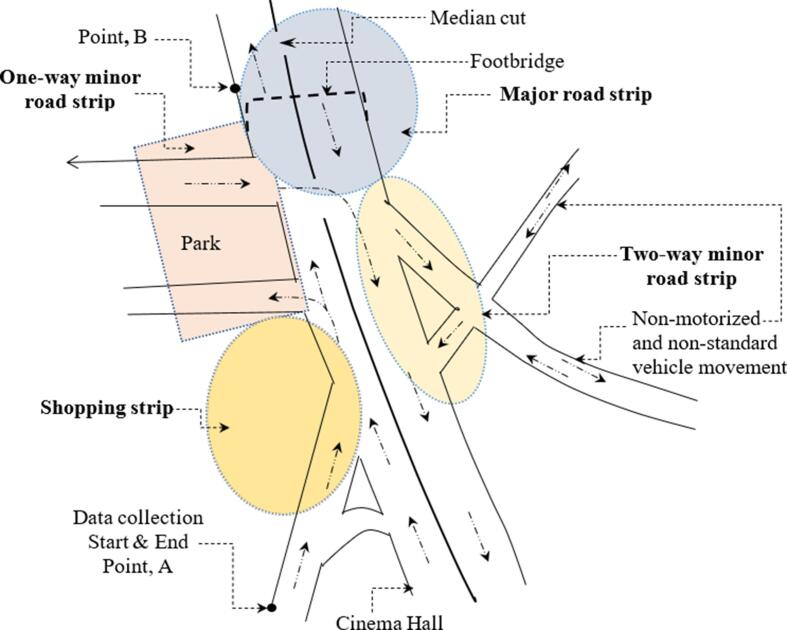
Think Aloud Data Collection Route at Farmgate Intersection.

Farmgate intersection, in central Dhaka, has been identified as a collision blackspot by local collision investigating authorities due to the regular occurrence of fatal collisions. Morency and Cloutier (2006) defined a blackspot as a site where more than eight pedestrian collisions happen over a period of five years. From 2009 to 2014, 19 collisions took place at Farmgate intersection, of which nine involved one or more pedestrians (ARI, 2015). The intersection has also been highlighted in the popular media for being one of the most dangerous in Dhaka, particularly for pedestrians (Farhin, 2017). The area around the large intersection serves as a significant business hub, and is home to many government offices, educational institutions, and commercial and financial institutions, and thus invites high pedestrian activity. The study route selected is presented in Fig. 2, representing an area which spans around 350 m from south to north. For analysis purposes, the study area was divided into four strips, displayed in Fig. 2: shopping strip, one-way minor road strip, major road strip, and two-way minor road strip. The reason behind selecting these strips was their different characteristics; the shopping strip and the major road strip have a six-lane, divided road (i.e., with median barrier), the one-way minor road strip is a two-lane road, with both divided and undivided sections, and the two-way minor road strip is two-lane, undivided road with extended width. Additionally, the road strips differ in terms of the activities undertaken and their traffic characteristics. Strip length varied from 154 m (shopping strip) to 285 m (two-way minor road strip).

### 3.3 Think aloud procedure

Participants were given a half-day training about the data collection method, the use of a GoPro camera (that was used to capture video and audio), and the process of verbalising their thought processes. Following desktop training, involving the use of video examples of individuals verbalising their thought processes, participants were taken to a small junction within a university campus where they performed several short, practical think-aloud sessions to become familiar with the process. The participants underwent several practice sessions until both the participant and the investigator were satisfied that the participant was able to provide continuous and concurrent verbalisations, without significantly impacting on their safety. After finishing the field training, all the participants returned to the training centre and a question-and-answer session was held to clarify their queries.

### 3.4 Data collection and analysis

Due to resource constraints, time limitations, and the availability of all participants at a time, data were collected on five different dates; however, the surveys were conducted at the same time and day of the week, and on days with similar weather conditions, in order to minimise differences in roadway and environmental conditions. Seven to thirteen participants participated in each day of data collection. Before beginning the trial, participants were given a brief on the study route. They were informed that they should start walking at one designated point near Ananda Cinema Hall (point A in Fig. 2), cross the road at a point near Khamarbari (point B in Fig. 2), and return to initial point after making a loop; however, they were instructed that they had full freedom to navigate around the intersection as they would normally do so. The forward view of the participant was recorded using a GoPro HERO5 camera attached to one of the straps of a backpack they were wearing. This also recorded the audio, capturing the participants’ verbalisations.

**Fig. 3:**
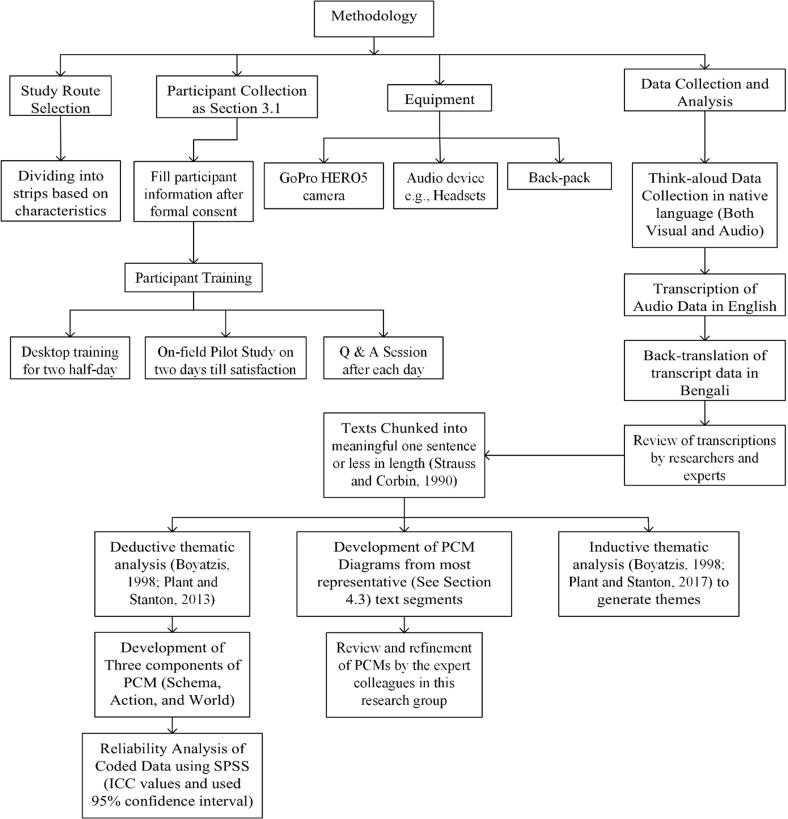
Flow diagram showing the step-by-step methodology.

The verbal protocols were provided in Bengali, the native language of the participants. Following verbatim transcription, all transcripts were translated into English. All of these translated transcripts were then back-translated into Bengali to check the accuracy of the translation, with any issues discussed and rectified by the research team. The transcripts were then segmented following the guidelines provided by Strauss and Corbin (1990). In order to maintain uniformity, all segmentation was done by one of the authors. Two additional authors also independently segmented 12 of the 46 transcripts in order to check for discrepancies. Any discrepancies were resolved collaboratively through discussion. Once segmentation was complete, each segment was assigned to one of the three categories specified in the PCM, namely schema, action, or world. Typically, 20% of samples are used for reliability analysis in PCM studies (e.g., Plant and Stanton, 2016). The segmentation process resulted in the 46 transcripts being divided into a total of 9752 segments; in order to determine the reliability of the PCM coding, 2638 of these text segments were selected randomly for coding by two additional individuals, representing more than 25% of the data. The intra-class correlation coefficient (ICC) estimates and their 95% confidence intervals were calculated using SPSS based on a mean-rating (k = 3), absolute-agreement, 2-way mixed-effects model. Percentage agreement score of 80% and above is accepted as good inter-rater reliability (Jentsch and Bowers, 2004). For all the pedestrian participants, data were collated into frequency counts for each of the three categories of schema, action, and world for the complete route and for all road strips individually. The step-by-step detailed methodology followed in this research is presented as a flow diagram, in Fig. 3.

## 4 Results and analysis

### 4.1 Reliability analysis

The number of segments into which each transcript was divided ranged from 113 to 408 (mean = 219.28, SD = 61.56). Word counts varied from 839 to 3526 (mean = 1763.28, SD = 585.29), and the time it took the participants to complete their journey around the study area ranged from 10 to 36 min (mean = 17.30, SD = 5.70). Words spoken per minute ranged from 55.93 to 147.15 (mean = 104.31, SD = 25.35). Regarding the reliability analysis, based on 27.05% of the total data (2638 of the 9752 segments present in the 46 transcripts), the intra-class correlation coefficient (ICC) was 0.988 (95% CI: 0.847 to 0.991), indicating excellent agreement between raters. The percentage agreement between author one and two was 92.30% (SD = 11.62), and was 91.17% (SD = 15.39) between author one and three, signifying an excellent level of agreement.

### 4.2 Code frequencies

The number of schema, action, and world coded segments across all 46 transcripts for the complete study route, and the four sections of the route individually (shopping strip, one-way minor road strip, major road strip road, and two-way minor road strip) are presented in Table 1. For all road sections and across all transcripts, the majority of text segments were coded as world, followed by action, and then schema. This pattern of these results mirrors that which has been found in previous PCM research (e.g., Plant and Stanton, 2016).

**Table 1:** Total and average frequency of schema, action, and world segments across all 46 transcripts.

	Schema		Action		World	
	Frequency (percentage)	Average per transcript (SD)	Frequency (percentage)	Average per transcript (SD)	Frequency (percentage)	Average per transcript (SD)
Complete route	1697 (17.40%)	36.89 (21.87)	2768 (28.38%)	60.17 (24.32)	5287 (54.21%)	114.93 (37.85)
Shopping Strip	537 (15.84%)	11.67 (6.84)	907 (26.75%)	19.72 (10.55)	1947 (57.42%)	42.33 (13.63)
One-way minor road	397 (18.60%)	8.63(5.92)	651 (30.51%)	14.15 (7.11)	1086 (50.89%)	23.61 (13.03)
Major road strip	405 (19.66%)	8.80 (8.42)	620 (30.10%)	13.48 (10.06)	1035 (50.24%)	22.50 (16.11)
Two-way minor road strip	358 (16.52%)	7.78 (7.27)	590 (27.23%)	12.83 (7.77)	1219 (55.25%)	26.05 (16.36)

### 4.3 Perceptual Cycle insights into pedestrian behaviours

**Fig. 4:**
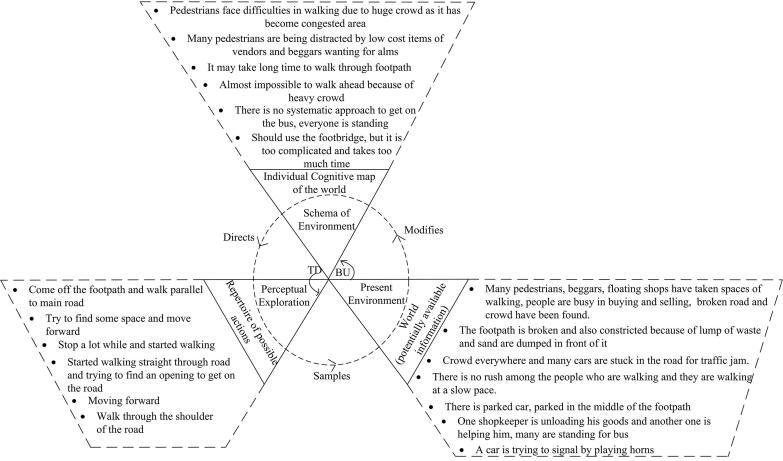
Amalgamated Perceptual Cycle Model of participants in the shopping strip.

**Fig. 5:**
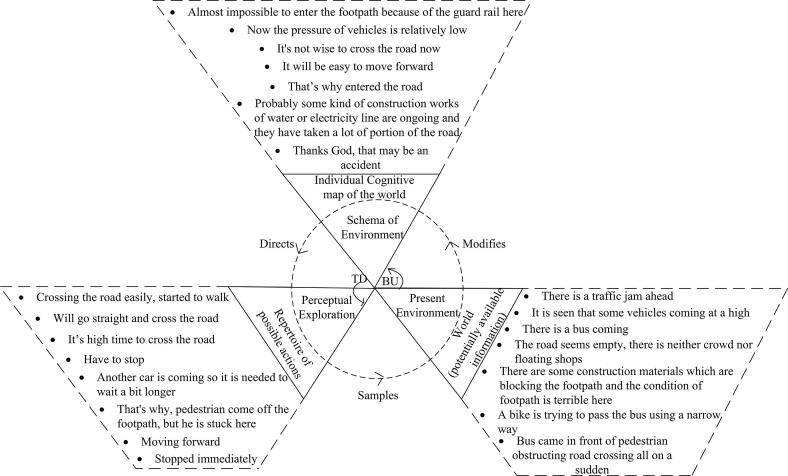
Amalgamated Perceptual Cycle Model of participants in the one-way minor road strip.

**Fig. 6:**
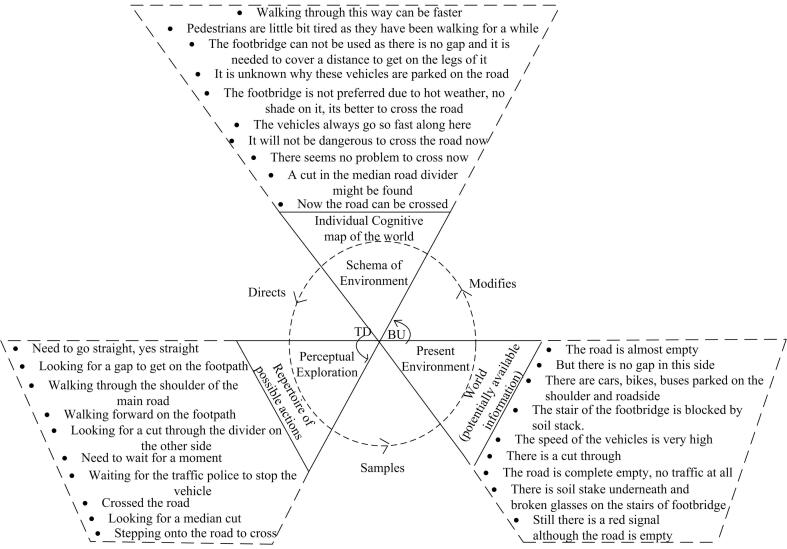
Amalgamated Perceptual Cycle Model of participants in the major road strip.

**Fig. 7:**
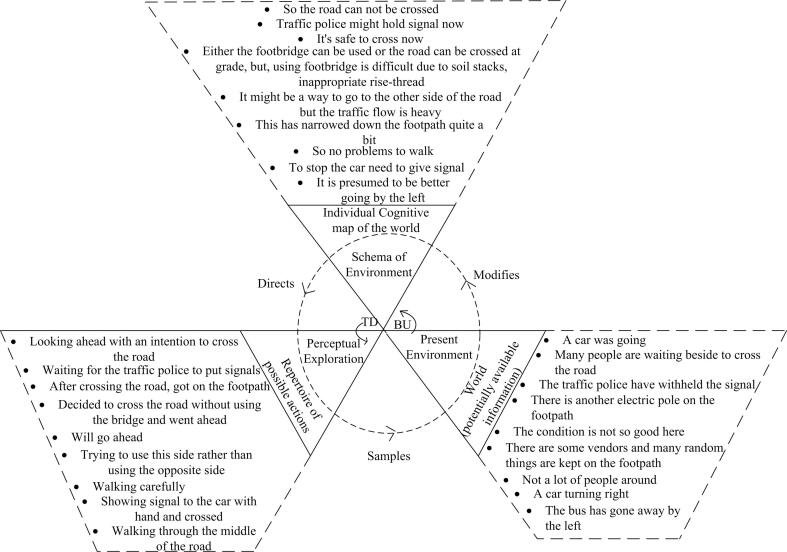
Amalgamated Perceptual Cycle Model in the two-way minor road segment strip.

Amalgamated PCMs were created for each of the four road sections in order to demonstrate the generalised decision-making processes in each area (see Fig. 4, Fig. 5, Fig. 6, Fig. 7). Developing amalgamated PCMs follows the approach used by Plant and Stanton (2016) when they produced representative PCM case studies of critical collision data to investigate aeronautical decision making. Data were included in the PCMs that were most representative, i.e., said by multiple participants (as agreed by the data coders). The representative text segments were chosen to be included in the PCMs by counting the frequency of data (e.g., number of text segments, word counts, words spoken per minute) and employing an inclusion / exclusion criterion e.g., include the ones that fall above the mean. PCMs for each of the road sections have been annotated with representative text segments below.

The amalgamated PCM of participants in the shopping strip is presented in Figure 4. Overall, the transcripts of the pedestrians in the shopping strip region highlighted the activities of vendors selling different items, and the large presence of beggars, with pedestrians being distracted by both. Such distractions have the potential to contribute to the incidence of vehicle–pedestrian collisions. The PCM analysis highlighted that pedestrians are constantly developing schemata based on present information and are changing their course of action accordingly. For example, **“***Many pedestrians, floating shops have taken spaces of walking, people are busy in buying and selling, broken road and crowd have been found. This footpath is also constricted because of lump of waste and sand are dumped in front of it* [world]. *Come off the footpath and walk parallel to main road* [action].**”** This gives an idea of why pedestrians walk on the roadside instead of the footpath, a very common occurrence across Dhaka; the environment compels pedestrians to change their course of action.

The PCM diagram in Fig. 5 summarises the participants walking along the one-way minor road section and shows the interaction between the pedestrian’s schemata, the available world information, and their actions. For example, participants have been found quoting, “*It is seen that some vehicles coming at a high speed*”, representing world information and a reason not to cross the road; *“It’s not wise to cross the road now*”. Thus, the schemata, and the information available, guided the pedestrian’s action of not crossing the road. Similarly*,* the quote *“There* [is] *neither crowd nor floating shops”* refers to available world information, resulting in the verbalised thought that *“It will be easy to move forward*”. This schema has developed through experience, i.e., knowing it will be easy to move because of the lack of crowds and shops; consequently, this informs the pedestrian’s action; *“Will go straight and cross the road*”. This behaviour is indicative of most pedestrian behaviour in the area; people do not wait for the signal of the traffic police, rather whenever they get the opportunity, they start crossing the road.

The amalgamated PCM diagram in Fig. 6 is for the major road strip, again drawn from the transcript of participants. The general themes at this section of the road related to wanting to cross the road quickly. This desire to cross the road quickly led to pedestrians to walk along the shoulder of the pavement, in order to quickly take advantage of small gaps in traffic. This desired action was hindered, however, by the presence of bikes and other vehicles parked on the shoulder. As such, participants walked along the side of the main road, off the footpath. Participants also commented that the footbridge was unsuitable for use due to the presence of a soil stack at the entrance and the fact there is no shade on it. Thus, road crossings were made using an informal (i.e., not purposely implemented) median cut (i.e., a gap in the central barrier in the middle of the road), despite the constant presence of high-speed motor-vehicles. This is highly typical of crossing behaviour at that road section, and across Dhaka, so the findings from this specific area should also be generalisable to the wider Dhaka region and across Bangladesh in general (i.e., the lack of shade over foot bridges and the presence of informal cuts in median barriers being common).

The amalgamated PCM diagram of Fig. 7 again shows the inter-relations between schema, action and world for all participants manoeuvring along the two-way minor road strip. It can be observed that the presence of temporary shops and other obstacles, such as construction materials and people waiting on the footpath made it so constricted that pedestrians, particularly those in a hurry, preferred not to use it. In addition to obstacles, the participants also referred to the quality of the footpath, citing its poor condition, the presence of electric posts, and the bad positioning of the entry and exit legs of the pedestrian footbridge. All factors influenced their decision not to use the footpath. Subsequently, as people are already walking on the road, they prefer not to get onto the footpath in order to use the footbridge to cross the road, rather they look for gaps in traffic, or police signals, crossing the road in what can be interpreted as a risky fashion. This phenomenon is very common for pedestrians across Dhaka city.

The PCM diagrams presented in Fig. 4, Fig. 5, Fig. 6, Fig. 7 show a number of similarities and differences across the four road strips. Similarities in world statements showed all strips to suffer from narrow and blocked or obstructed footpaths in poor condition, as well as from heavy pedestrian volumes. These led to similar schema statements expressed across all road strips, i.e., that pedestrians face difficulties and feel tired while using the footpaths. As a result, pedestrians perform the action of coming off the footpath and stepping onto the roadside; this was noted in all road strips. When on the roadside, pedestrians mentioned needing to walk carefully and to continuously look for opportunities to get back on the footpath, as these opportunities are few and irregular. It is also worth noting that road users’ behaviour is affected by the presence of law enforcement; this was also common to all strips. In terms of the differences, pedestrians in the shopping strips were found to be slower than in the other strips. This is due to especially heavy pedestrian volume in that area, the many distractions to walking that are present, including the non-standard use of footpaths. Activities such as the boarding and alighting of public and private transport passengers, uncontrolled parking, and other non-standard uses of the footpaths were found to be influential in the shopping strip and two-way minor road strip, whereas high speed vehicles were found to be influential in the major road strip. Verbalisations made in the one-way minor road strip featured less reference to stopping distances than in other strips. At the major road strip, the road can be crossed either by using a footbridge or by a cut through the median; however, using the footbridge is difficult due to the presence of soil stacks, the faulty rise-tread ratio of the stairs, and unfavourable weather. At the one-way minor road strip there is no footbridge, hence this was not mentioned by participants while in this section. At the major road strip, pedestrians cross the road following the hand signals of traffic police; however, at the one-way minor road strip, pedestrians cross the road whenever they get chance, often by using their hand to signal the vehicles to stop, as the presence of traffic police is much less common than it is at the major road strip. Table 2 summarises the key findings from the PCM analysis of pedestrian behaviour and presents a number of recommendations as to how safety could be enhanced.

**Table 2:** Summary of PCM analysis of pedestrian crossing behaviours and safety recommendations.

Finding from PCM analysis	Impact on pedestrian safety	Recommendation
Mental discomfort caused by cyclists on the footpath	Creates discomfort for the pedestrians in an already constricted and crowded footpath	Have public safety campaigns/educate cyclists to make people aware of this issue and the safety implications
Negative/shortcut attitudes of pedestrians	Although there is a footbridge in front, they may not use it for crossing the road; absence of enforcement, and poor footbridge quality (and other attribute) encourages street-level crossing	Enforcement by police, education campaigns to create mass awareness about the safety benefits; strict punishment needs to be enforced for irregular/random risky road crossing and social awareness campaigns need to be undertaken
Waste on footpaths	Compels pedestrians to walk on active road instead of on the footpath	Resource the regular cleaning of footpaths
Undulating and constricted footpath	Pedestrians prefer to use road to walk on comfortably	Non-standard footpaths should be designed and constructed following standard guidelines (being a developing country, there is no established geometric design standards, however, project consultants mostly follow guidelines as per AASHTO, 2018, Manual, 2016 etc.) and regular maintenance must be ensured
Large poster / advertisement boards on footpath	Distracts pedestrian’s line of sight	No paper, posters or advertisement boards blocking the pedestrian’s line of sight should be allowed to be installed
Heavily crowded footpath	Huge contraflow of pedestrians, results in pushing by other pedestrians, encourages leaving the footpath	Pedestrian footpath should be widened to ensure satisfactory level of service
Inappropriate design, location, rise-tread ratio, wet, slippery footbridge	Pedestrians show less interest in using footbridge and instead cross at street level	No soil stacks and other wastes can/should be dumped on/around and beneath the footpath/bridge, resource regular cleaning of stairs; footbridge needs be located at an appropriate location/aligning pedestrian origin–destination lines and designed and maintained in a way that use is appealing
Presence of electric posts, pillars, vendors, floating shops on footpath	Constricts the footpath leaving little or no usable space for pedestrians	No posts, pillars, temporary and permanent shops should not be allowed on footpaths; conflicting use of footpath should be strictly enforced
No systematic approach to board buses	Everyone remains standing on footpath and makes the footpath crowded/ blocked	Designated places such as bus bays for the loading–unloading of passengers, with waiting facilities; instead of boarding in groups, queue discipline should be maintained complying first in first out (FIFO) based queue discipline
Rolling behaviour on road in road crossing/random crossing	Walk/cross the road in a zigzag pattern	Enforcement of law by enforcing agencies so that pedestrians follow traffic rules; picket railing/barrier should be in-place on footpath edge and as well as on the divider/median; signal should be made functional and pedestrian signal phase including all-red period should be included
Damaged footpath	Compels pedestrians to walk on active road instead of on the footpath	Develop a database of footpath faults and prioritise the worst ones for repair
Storage and dumping of construction materials on footpath	Constricts the footpath leaving little or no usable space for pedestrians	Introduce fines for construction workers who are caught putting construction materials on the footpath
Parking of vehicles on/around the footpath	Constricts the footpath leaving little or no usable space for pedestrians	Restriction on parking on/near the footpath; vehicles should be fined if they do not comply
No traffic or pedestrian signal	In order to cross the road, pedestrians are forced to stop vehicles by showing hand and crossing through group action	Provide a designated time and space for the movement/crossing of pedestrians
Following social norms	Risky road crossing behaviour encourages others to get involved in similar risky manoeuvres (peer influence)	Impose restrictions on between-vehicle crossing, and incorporate a dedicated signal time for pedestrians
Platoon action of pedestrians	Pedestrians force incoming vehicles to stop and cross the road in a platoon	Allow pedestrians to cross only when they get signal to cross
Disrespect for rules	Pedestrians do not follow any rules, they stop vehicles signalling by hand and start crossing roads	Appropriate and user-friendly design of footpath, regular cleaning of footbridge and safe footpath landing must be ensured; design should be inclusive and proportionate based on pedestrian flow demand and level of service

## 5 Discussion

This study of road user behaviour in Dhaka, Bangladesh, highlighted the difficulties experienced by pedestrians when attempting to navigate a busy section of the city. The participants’ verbal reports revealed various factors that act as barriers to safe crossing, e.g., the presence of the stores, hawkers, vendors, beggars, floating shops, and illegal activities that blocked and constricted the footpaths. Additional references were made to other impediments to pedestrian travel, e.g., the presence of construction materials like brick, gravel, sand etc.; poorly maintained, uneven, and broken footpaths; blockages due to the accumulation of rubbish and waste; the parking of vehicles on and near the footpaths; use of footpaths as a stand for buses, CNGs (small, motorised public transport vehicles) and other vehicles (without organised methods or places of boarding or alighting those vehicles); the presence of a police box leaving no space for pedestrians to walk; a large political banner blocking road users’ vision; and broken footpath barriers impeding normal pedestrian activities. Pedestrians in Dhaka are compelled to stop frequently and walk in a zigzag pattern, and to walk on the main road itself, due to the lack of space on footpaths. This undoubtedly contributes to the volume of pedestrian collisions that take place in the urban streets of Bangladesh.

The verbal transcripts also highlighted the problems caused by the highly raised nature of the footpaths (in terms of actually mounting and using them), and the inability to use the footbridge due to pedestrian guard rails blocking their path (where they had been walking on the road), and rubbish around the entrance of the footbridge. The stairways of the footbridge were also blocked, with the steps littered with broken glass, bricks, and soil stacks. Moreover, pedestrians reported that the stairs were not comfortable to use because of the uneven rise-tread ratio. Hence, in order to cross the road, instead of using the footbridge, pedestrians used the road, going over the median barrier where its height allowed, through or around the barriers if any holes were present, or through bars spaced far enough apart for them to pass through. On top of these factors, time saving was also cited as an influential factor in the non-use of the footbridge.

According to Kadali and Vedagiri (2016), six-lane, divided roads see fewer pedestrian vehicle conflicts, and therefore improve pedestrian safety due to fewer interactions with vehicles; however, severity is significantly higher when collisions do occur, due to the higher speeds. From the participants’ verbal reports, it was seen that the shopping strip (containing a six-lane, divided road) had the highest number of links, and a high number of text segments per minute indicating more interaction with the environment.

### 5.1 Environmental factors affecting pedestrian behaviour

The physical world has a large impact on a pedestrian’s understanding of a situation, hence their decision-making process. In low-income countries like Bangladesh, footpaths are often found not to conform to accepted international standards with respect to geometric and operational conditions; results from this study show just how influential such factors are on pedestrian movements. If the decision-making processes that lead to such use (or misuse / non-use) of infrastructure can be better understood, better treatments can be designed which will help increase not only the efficiency of pedestrian flow, but the safety of the overall system. Environmental modification, through the modification of road designs, without incorporating considerations of pedestrian decision-making (i.e., without designing for unpredictable pedestrian behaviours on the road), will likely fail. The verbal protocols drew attention to a number of different illegal activities taking place on and around the footpath, forcing pedestrians to move along the shoulder or the road; consideration for these must be incorporated in road layout designs that facilitate pedestrians’ crossing through designated places, free from impediment, whilst accommodating the commerce that will inevitably be present.

The transcripts made clear that pedestrians are compelled to take risky decisions and perform risky behaviours. Pedestrians look for short-cuts, often manifesting as informal crossing points. Although some participants explicitly stated that using the footbridge is safer than crossing at road level, eventually they intentionally avoided using the footbridge. Such violation of rules is not consistent; for example, participant 8 referred to footbridge avoidance; however, the very same pedestrian followed rules when in the presence of law enforcing agencies. This perhaps gives an indication of the importance of enforcement in the Bangladesh road context. Distracted walking behaviours were also revealed in the transcripts, caused by excitement arising from the occurrence of a collision, the presence of floating shops and their items, nearside park activities, and so on. Barton et al. (2016) posited that pedestrians engage in riskier behaviours during crossing if they are distracted. The participants also showed acceptance of small margins of safety when finding or estimating a suitable safe gap (as was said by Zhuang and Wu, 2012), often resulting in the need to run to complete the crossing. This is a significant contributor to collisions (Avinash et al., 2019).

### 5.2 Impact of pedestrian attitudes and behaviour on road safety

According to Avinash et al. (2019), many collisions occur due to negative attitudes and non-compliant pedestrian behaviours. This also came out in the transcripts; however, positive behaviours were also reported, such as waiting for a hand signal of the traffic police officer to stop the vehicles before crossing, or waiting for a low-risk opportunity to cross, being watchful, using footbridges, etc. It was also found that most of the pedestrians showed tendencies to form crossing platoons, i.e., crossing with others or waiting with others. This pedestrian platoon action sometimes (especially during rush hour) disrupts the normal traffic flow, keeping the vehicles waiting until all the pedestrians in the platoon cross. This can induce drivers to speed up (while in hurry) in order to pass before the platoon blocks his or her path. Such driver behaviours can lead to collisions, due to the sudden speed increase. Although Avinash et al. (2019) argued that larger pedestrian crossing platoons help people to feel safe while crossing, the behaviour can be dangerous, as a pedestrian may blindly follow other road users, whether safe or not.

A number of participants showed ‘rolling behaviour’ (defined by Brewer et al., 2006, Kadali and Vedagiri, 2016), where the pedestrian uses gaps between vehicles to cross, moving in a zigzag pattern. According to Avinash et al. (2019), this rolling behaviour arises during periods or areas of heavy traffic, something typical of the Farmgate study area. This rolling behaviour reduces safety margins and increases vehicle–pedestrian conflicts (Kadali and Vedagiri, 2016); however, this is perceived as necessary if one is to cross the road, and is expected by drivers, many of whom yield to the crossing pedestrians. This situation is quite common in low-income countries like Bangladesh.

### 5.3 Impact of travel speed and time on decision-making

Regarding speed of travel, a number of participants referred to moving slowly due to the presence of activities on the footpaths. Consequently, footpaths become highly crowded, further reducing pedestrian travel speed. While crossing, however, slow speeds can be dangerous, especially when crossing informally in fast moving traffic (Lobjois and Cavallo, 2007, Oxley et al., 2005, Kadali and Vedagiri, 2016).

A number of participants mentioned long waiting times (i.e., when waiting to cross) mostly caused by heavy and continuous traffic. These higher waiting times led the participants to make risky crossing decisions; pedestrians mentioned the (perceived) need to take any crossing opportunity that arose, as otherwise they would not be able to cross at all. This forces the pedestrians to accept smaller vehicular gaps. According to one study, pedestrian waiting times of more than 30 s leads to riskier crossing behaviours (Kaiser et al.,1994). The verbal protocols highlighted that path blockages, due to parked vehicles on and around the footpath, also led to dangerous and risky decisions; this supports the findings of Demir et al. (2019) that parked cars impel pedestrians towards violation and high-risk walking behaviours. Regarding the estimation of vehicle speed, this is especially complex in Dhaka (and other low-income settings) due to the presence of a large variety of standard and non-standard motorised and non-motorised vehicles. These have a wider variety of potential speeds, something that leads to ambiguous decision making as to whether to cross or not, as speed helps pedestrian decision making (Sun et al., 2003).

### 5.4 Lack of discipline and infrastructure leading to unsafe pedestrian behaviour

Non-standard vehicles, particularly public service vehicles (e.g., rickshaw, CNG, etc.), often do not (or cannot) follow the rules and regulations of the roads (e.g., no indicator lights, coming suddenly from the wrong direction, stopping haphazardly causing congestion and obstructing the movement of other vehicles); this was verbalised by a number of the participants. References were also often made to the inadequacy of road furniture and infrastructure, for example to the inappropriate height of curbs and steps, or to narrow and slippery paths and footbridge steps, or to their inappropriate placement along the road. According to Demir et al. (2019), pedestrian crossings or overpasses should match the pedestrians’ desirable walking route, otherwise they will not be utilised. Furthermore, the main road strip is characterised by the presence of relatively high-speed vehicles and relatively few pedestrians, and relatively few illegal activities (like temporary shops) are found on the footpath. These factors, in combination with the presence of a nearby police box, may have a reducing effect on erratic pedestrian movements. This points to the influence of law enforcing agencies on pedestrian behaviour, both directly, and indirectly through its influence on footpath-based illegal vendor activities.

### 5.5 Schema influencing pedestrian behaviour

Using the PCM as a framework for the analysis revealed that participants referred less to their schema (i.e., things they were thinking about, past experiences and expectations) than they did to world information or the actions they took (see Table 1). This does not necessarily indicate that considerations of schema were less present, rather it may be due to the schema element being the hardest of the three to elicit and measure (Plant and Stanton, 2013, Plant and Stanton, 2016). The results shown in Table 1 regarding less schema frequency is comparable to that of Plant and Stanton (2016). They studied the decision-making processes of pilots and found that there were fewer references to schema, compared to action and world, as this type of information is often inaccessible given the automated way people interact with the world. This is typical in situations involving high levels of expertise, where behaviour becomes almost automated, i.e., when thinking and working does not require significant time or effort (Jhangiani et al., 2014, O'Hare et al., 2009). Our study represents such a situation, as all the pedestrians were familiar with the road environment under study (and with the act of being a pedestrian). In addition, this automatic behaviour could be dangerous for the pedestrians while crossing roads, as automatic behaviour involving less conscious attention in the decision-making process may lead to the erroneous identification of vehicular speed and the acceptance of unsafe gaps. The resulting hazardous crossing is, according to de Lavalette et al. (2009), implicated in the majority of pedestrian injuries. In addition, when a pedestrian encounters a piece of world information, a schema is triggered by that information; however, they may not change their schema based on the world information. Thought processes can be so well learned and proceduralised that they are no longer available for conscious introspection, hence are not available to be verbalised. This bias, towards world and action statements (that are more easily verbalised), has also been discussed by Annett (2002). Moreover, this bias may arise from a tendency to be overconfident in one’s own skills, abilities, and judgments (Jhangiani et al., 2014). Due to overconfidence, pedestrians have a reduced awareness of their limitations, thus leading to erroneous road maneuvering and, consequently, to collisions. This can be problematic; for example, if a pedestrian thinks that no vehicle usually comes from the wrong direction (violating laws) while crossing road, when such an occurrence does happen (which is not altogether uncommon in Dhaka), a serious collision may occur.

## 6 Limitations and future scope of study

The main limitation of this research is the non-representative nature of the sample. Although the sample is mostly representative of the study area, with young students representing the dominant pedestrian group found in the Farmgate area (due to the presence of a large number of educational institutions), it is not representative of the wider Dhaka population. As such, future research would do well to investigate the decision-making processes of pedestrians of different demographic characteristics, particularly older pedestrians. Similarly, and also due to sample resource constraints, the sample was weighted towards males. Again, this reflects the on-road, pedestrian reality in Dhaka; however, if we are to achieve gender equity in society, we must also strive for gender equity in research. We accept this as a limitation of the research. Additionally, only one intersection was selected for the setting in which to conduct this research. Although this intersection was identified as the most hazardous location for pedestrian collisions in Dhaka, future research should extend the approach to different road environments, including rural settings. This would widen our understanding of pedestrian behaviour in Bangladesh. Moreover, participants acquainted with the intersection were recruited for this study for extracting natural pedestrian behaviour. So, it is expected that the experiment will yield similar results if it is to be repeated with the same participants. However, it may vary slightly depending on the mental state of the participant and environmental condition he or she is facing. The extent of this behavioural variation of same participant in different mental state can be a future scope of research. The future work would also do well to compare detailed collision statistics with qualitative user experience, however it is beyond the scope of this study, as it would involve deep analysis of any collision reports. Furthermore, our focus has been only on pedestrians; widening the lens to include multiple types of road users would also contribute to a deeper, more holistic understanding of decision-making and behaviour differences in the complex road scenarios of low-and middle-income countries.

## 7 Conclusion

The current study approached pedestrian road safety through investigating pedestrian decision-making in an urban setting in Bangladesh. From the analysis of the verbalised thought processes of 46 pedestrians, diagrams were developed based on the Perceptual Cycle Model distinction of schema, action, and world. In the complete route, and across the four distinct sections of that route, the pattern of schema, action, and world were the same; world and action statements were far more common than references to schema, suggesting the existence of automatic cognition and behaviour, which can lead to faulty decision making, insofar as conscious attention is lacking. In a complex environment such as Dhaka, this can lead to erroneous decisions, or the erroneous identification of environment characteristics, which in turn can lead to collisions. Pedestrian decision-making is guided by many factors, with the pedestrians’ behaviour being shaped by the environment and their past experiences. From the verbal reports gathered in this study, it was found that pedestrians are compelled, by the environment, to perform risky behaviours. It was also found that negative road safety attitudes existed, with many participants habitually breaking pedestrian rules. Perhaps most importantly, pedestrians considered the rules and regulations to be for drivers. That being said, pedestrians did follow rules in the presence of an enforcement officer; this has implications for effective system design.

Pedestrian decision-making and behaviour must be incorporated into the road system design process. Through understanding these phenomena, we can better design the road safety system (through training, infrastructure, or beyond) in order to minimise road trauma. By building our understanding of pedestrian decision-making processes in complex environments, particularly in low- and middle-income settings where such research is least undertaken yet most needed, we can inform the design of interventions that will improve pedestrian (and wider system) safety and efficiency.
